# Editorial: The impact of climate change on allergic disease

**DOI:** 10.3389/falgy.2023.1246899

**Published:** 2023-10-19

**Authors:** Juan Aguilera, Gabriel Ibarra-Mejia, Mary Johnson

**Affiliations:** ^1^ Center for Community Health Impact, The University of Texas Health Science Center at Houston School of Public Health, Houston, TX, United States; ^2^ Department of Public Health Sciences, The University of Texas at El Paso, El Paso, TX, United States; ^3^ Department of Environmental Health, T.H. Chan School of Public Health, Harvard University, Boston, MA, United States

**Keywords:** climate change, allergic disease, pollen, immune system, air pollution, public health, environmental stressors, global health

**Editorial on the Research Topic** The impact of climate change on allergic disease

## Exploring new insights and challenges

**Figure 1:**
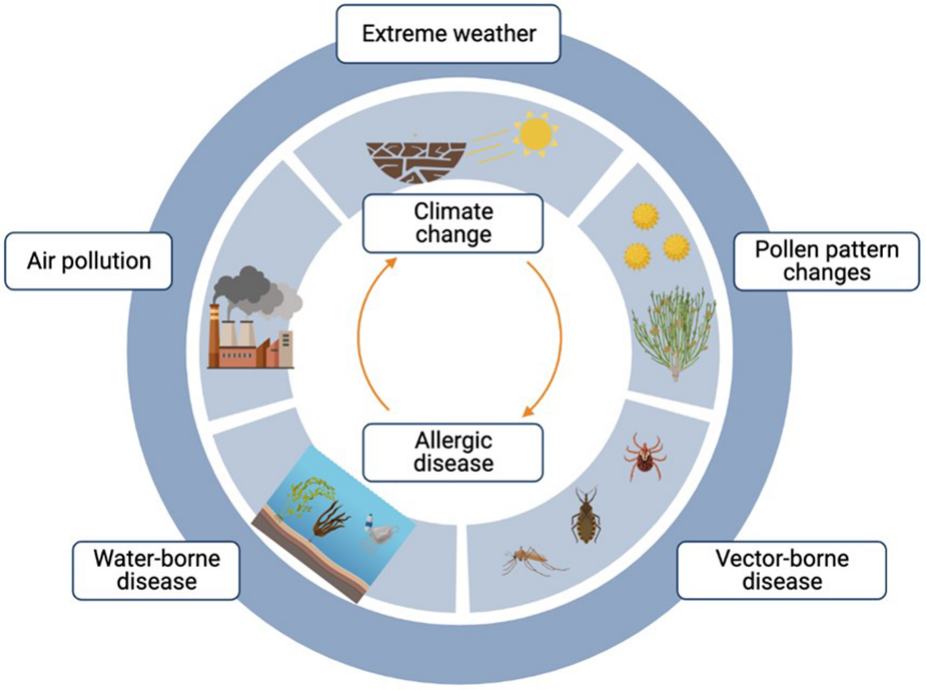
Current evidence and climate models show that an increasing global temperature will intensify extreme weather events around the globe, thereby amplifying allergic diseases.

Climate change poses a significant threat to human health, and its impact on allergic diseases is an area of growing concern. As global temperatures rise and weather patterns shift, the distribution and abundance of allergenic pollen and the length of pollen seasons are expected to change, leading to increased allergen exposure and exacerbation of allergic diseases, as shown in Figure 1 (1–3). In this Research Topic we present articles that delve into how climate change influences the incidence and severity of allergic diseases, offering valuable insights into this complex relationship and shedding light on potential strategies to mitigate the impacts on human health.

A significant challenge in managing allergic diseases is pinpointing the allergens responsible for triggering allergic reactions (4). Pollen, a substantial source of allergens, is influenced by climate change, affecting the quantity and quality of allergenic proteins (5–7).

In their article, Juprasong et al. consider the effects of environmental stressors—flood, salt, and drought—on the expression of β-expansin, a major pollen allergen, in rice and maize (*Oryza sativa* and *Zea mays*, respectively) pollen. Their study focuses on two specific β-expansins, OsEXPB in rice and ZmEXPB in maize, which play essential roles in cell wall modification and growth processes. The research sheds light on how these stressors can influence the expression of β-expansin and, consequently, impact allergic reactions, particularly in relation to grass group-1 pollen allergens. These findings highlight the importance of understanding the specific environmental factors influencing allergenicity to better predict and manage climate change impact on allergic diseases.

Another crucial factor in allergic disease development is the role of environmental factors on the immune system. Exposure to air pollution, for instance, has been linked to increased susceptibility to allergic sensitization and exacerbation of allergic diseases (2, 8). On this topic, Liu et al. developed an experimental ragweed pollen (RWP) allergy model in mice to investigate how the number of RWP and the geographical origin of RWP influence allergic disease. The findings revealed that environmental factors impact RWP and result in a spectrum of allergic lung diseases, which may contribute to the increasing sensitization rates and severity of pollen-induced disease exacerbations in patients. This research highlights the necessity of a multidisciplinary approach to addressing climate change's impact on allergic diseases, calling for collaboration among immunologists, allergists, environmental scientists, and public health experts to develop comprehensive strategies for prevention and mitigation.

As climate change advances, alterations in the distribution and prevalence of allergenic pollen across regions are anticipated, potentially affecting millions of people suffering from pollen-induced allergic diseases (9, 10). Ren et al. developed a modeling system to simulate the spatiotemporal distributions of major pollen aeroallergens, such as, oak, and ragweed pollens across the contiguous United States for historical (2004) and future (2047) conditions. The results showed substantial regional variability in pollen metrics, with ragweed pollen seasons starting earlier and lasting longer in all climate regions and varying timing and magnitude of oak pollen seasons. These findings highlight the influence of climate change on airborne allergenic pollen exposure and emphasize the need for proactive measures to mitigate climate change's effects on human health.

Lastly, Singh and Kumar offered an overview of the multifaceted ways climate change is projected to impact allergic diseases, supplementing a broad spectrum of scientific literature with calls to action (11, 12). Their article delves into the complex and discusses the link between climate change, the increased prevalence and severity of asthma, and related allergic diseases, highlighting how greenhouse gases and rising temperatures impact pollen release patterns. Altered pollen patterns can exacerbate respiratory allergies in susceptible individuals. Anticipating future allergic disease burdens can help public health agencies develop strategies to mitigate the health challenges expected in the coming years. Furthermore, the article underscores the need for interdisciplinary collaboration to tackle the challenges posed by climate change and allergic diseases.

In closing, these articles offer valuable insights into how climate change influences the prevalence and severity of allergic diseases. By bringing together experts from various fields, such as allergists, immunologists, environmental scientists, and public health specialists, a holistic understanding of the issue can be achieved, allowing for the development of more effective and targeted strategies to mitigate the health risks associated with climate change and allergens. The findings emphasize the urgency of minimizing climate change's effects on human health. Proactive measures, such as reducing carbon emissions, devising new strategies for managing allergic diseases in a changing climate, and increasing public awareness of climate change's health risks, are vital. These articles significantly contribute to our comprehension of climate change's impact on allergic diseases and inform the development of strategies to mitigate its effects on human health.
